# Organisational culture and ethnic diversity in nursing homes: a qualitative study of healthcare workers’ and ward nurses’ experiences

**DOI:** 10.1186/s12913-022-08184-y

**Published:** 2022-06-30

**Authors:** Jonas Debesay, Sanjana Arora, Marit Fougner

**Affiliations:** 1 Faculty of Health, Department of Nursing, Oslo Metropolitan University, PO box 4 St. Olavs plass, NO-0130 Oslo, Norway; 2 Faculty of Science and Technology, Department of Safety, Economics and Planning, University of Stavanger, PO box 8600, 4036 Stavanger, Norway; 3 Faculty of Health, Department of Physiotherapy, Oslo Metropolitan University, PO box 4 St. Olavs plass, NO-0130 Oslo, Norway

**Keywords:** Discrimination, Ethnic, Inclusion, Manager, Multicultural, Organisational culture

## Abstract

**Background:**

The increase in care needs that comes with an ageing population, in combination with a shortage of healthcare workers, has made ethnic diversity among healthcare workers (HCW) an evident reality across many countries. This article aims to explore how a multicultural workplace is experienced, through the accounts of HCWs and leaders in nursing homes.

**Methods:**

This article reports on the findings from qualitative interviews with 16 HCWs and managers from nursing homes in Oslo. The interviews were conducted from August to September 2021. We analysed the data using a reflective thematic analysis informed by a hermeneutic-phenomenological approach.

**Results:**

Six themes emerged from the interview data: (1) understanding diversity through shared norms and multicultural experiences, (2) greater flexibility in a multicultural workforce, (3) challenging traditional norms in a multicultural workforce, (4) language proficiency and exclusionary practices at work, (5) perceptions of the role of the ward nurse, and (6) prejudices among and harassment from patients.

**Conclusions:**

To ensure the effective organisation and wellbeing of HCWs in a multicultural workforce, managers must develop an inclusive organisational culture. They must be able to engage with difficult topics and conflicts that may arise in the working environment.

## Background

With the ageing of the population across the world [1], the question of rising needs for care has become of paramount importance in many countries, including Norway, where the ageing of the population along with high life expectancy means that there will be a large proportion of older adults in need of professional care in the future [2]. In recent years, nursing homes in Norway have assumed greater responsibility for a larger number of older patients, as well as for more patients with higher needs [3]. Along with predictions of shortages of healthcare workers, care services for the elderly are one of the biggest future challenges in Norway [2, 4, 5].

To meet the rising demands of elderly care, and in line with increasing globalisation, ethnic diversity among healthcare workforces has become an evident reality across many countries, including Norway. In 2021, approximately 18% of the population of Norway had an immigrant background [6]. Healthcare services is the sector that employs the highest proportion of people with an immigrant background [7], and many of them work in nursing homes [8]. Nursing homes have thus become increasingly culturally diverse [9–11]. Immigrants make up an increasing proportion of municipal care workers, accounting for over 17% of the workforce in 2020. They come from different parts of the world, with the largest group coming from Asia, Eastern Europe in second place and Africa in third place [12].

Ethnic diversity has thus become a significant component of the nursing healthcare workforce in Norway. A few studies have investigated how employees with immigrant backgrounds have experienced challenges in municipal services [9, 10, 13, 14]. Others have looked at the benefits of recruiting more people and often point out that a multicultural workforce can contribute to increased access to higher-quality care services for the entire population [15, 16]. However, we still know little about how diversity is perceived and experienced from the perspective of both ethnic minority and majority healthcare workers (HCW) in the everyday organisational culture of nursing homes. Therefore, in this article, we have explored how a multicultural workplace is experienced through the accounts of HCWs and leaders in nursing homes.

### Multicultural workforce and organisational culture

It has long been established that a diverse workforce can be a strength in healthcare; however, it must be managed in a good way to achieve the goal of good quality and equitable healthcare services for all [17]. According to Thomas [18], the concept of diversity refers to differences and similarities that are put together in a complex and sometimes tensioned context. These similarities and differences can be about gender, ethnicity, sexual orientation or age. Thus, diversity is a complex concept [19]. In this article, we focus on ethnic or multicultural diversity among healthcare workers in nursing homes.

Organisational culture is an important context for understanding diversity experiences among workers situated in institutional practices [20]. According to Schein and Schein [21], organisational culture is a framework of basic assumptions discovered or developed by a particular group in learning how to deal with its problems through external adaptation or internal unification.

Organisational culture can be conceptualised in three components which manifest themselves at different levels of observability [21], which are (1) artefacts, (2) espoused values, and (3) basic assumptions. Artefacts represent the highest level of cultural manifestation. These include the symbols, structures and rules of an organisation that are openly visible to everyone. On the second, less concrete, level are the espoused values either that people have adopted or that serve as norms to be followed. Espoused values are reflected through conscious strategies, goals and philosophies, and they function as guidelines in doubtful and difficult situations. The least observable and final level of the culture are the basic assumptions and values which operate largely at a subconscious level. The basic assumptions are relevant in understanding structures and predicting behaviours [21], such as those towards ethnic diversity in organisations.

Furthermore, assumptions help groups function and survive during external adaptation and internal integration. In the context of our study, external adaptation implies meeting the expressed goals and values of the organisation, i.e., the effective delivery of healthcare services to patients/residents. Internal integration refers to how the workforce organises and maintains itself as a group, for example, through group boundaries, criteria for inclusion and exclusion, power distribution and unexplainable events [21].

Therefore, to understand how organisations and the workforce relate to ethnic diversity and multiculturalism, an exploration of organisational culture is pertinent. Exploration of diversity experiences reflected through the artefacts, espoused values and basic assumptions can thus provide knowledge about (a) how diversity ideas are both perceived and practised within nursing homes and (b) the implications of these on the internal and external functioning of the organisation.

### Nursing homes in Norway

In Norway, nursing homes are designed for residents who require a high degree of medical care and assistance with daily activities. There are also short-term rehabilitation departments within some nursing homes for patients to stay after hospital discharge. Provision of care in nursing homes is regulated by “Regulation of quality of care”. This implies that the basic needs of the residents’ such as psychological needs, preservation of dignity and self-respect, the degree of choice within the daily routine, physical needs (including nutrition), and social needs, should be satisfied [22, 23].

Nursing homes are funded from local and national taxes and user charges. The co-payment level by the residents is related to income and as such they typically pay between 75% and 85% of their income to the municipalities. Most nursing homes are owned by the municipalities. There is also a small percentage of nursing homes owned and managed by voluntary organisations, but where care is financed by the municipalities. In addition, there are also few commercial homes where residents pay full fee. The eligibility criteria for nursing homes are set by the municipalities. It is based on an assessment of needs, which include recommendations from the general practitioner of the patient. Decisions are taken by the provider or by an independent unity with the municipality’s health care system [24].

## Methods

This study is based on a hermeneutic-phenomenological approach [25, 26]. We were interested in the subjective experiences of understanding HCWs and ward nurses in a multicultural workforce in municipal and private nursing homes in Oslo.

### Recruitment and study participants

To recruit study participants, we approached The Nursing Home Agency and presented the study’s area of interest with topics and questions introduced in an information letter. The agency administration forwarded this letter to relevant nursing homes, which helped recruit study participants. We recruited 16 participants from four nursing homes in areas with a higher proportion of minority HCWs and service users. In the nursing home wards, 50–70% of the HCWs had a minority background, while 10–15% of patients had a minority background. The participants included four ward nurses, who were all ethnic Norwegians. Of the 16 participants, two were male and fourteen were female. Seven of the nurses had an ethnic minority background, and the remaining were ethnic Norwegians. The HCW group consisted of nurses and auxiliary nurses (Table 1).

**Table 1:** Study participants

Participant	Gender	Age (range)	Country of birth	Occupation	Years as HCW/ward nurse	Years of residence in Norway
1	F	30–40	Central Asia	Nurse	12	21
2	F	30–40	South Asia	Aux. nurse	10	24
3	M	40–50	East Africa	Nurse	2	18
4	F	40–50	Central Asia	Nurse	13	22
5	F	40–50	Central Asia	Aux. nurse	4	22
6	M	40–50	West Africa	Aux. nurse	21	32
7	F	40–50	Central Asia	Aux. nurse	16	23
8	F	50–60	Norway	Aux. nurse	30	
9	F	50–60	Norway	Aux. nurse	22	
10	F	30–40	Norway	Nurse	13	
11	F	30–40	Norway	Nurse	12	
12	F	40–50	Norway	Ward nurse	12	
13	F	40–50	Norway	Ward nurse	5	
14	F	40–50	Norway	Ward nurse	6	
15	F	40–50	Norway	Ward nurse	6	
16	F	50–60	Norway	Ward nurse	4	

### The interviews

The data for this study draws from 16 semi-structured interviews which were conducted at the participants’ workplace from August to September 2021. The interviews were conducted by a senior researcher with extensive experience in research on municipal healthcare and migration. He is a trained nurse, a male, and an immigrant from Africa. We developed a semi-structured interview guide to explore the participants’ perspectives on and experiences of working at and/or managing an ethnically diverse organisation, their perceived benefits and challenges of working in an ethnically diverse organisation, ways in which they utilise such diversity, and the need for further support in this regard. Each interview lasted approximately 60 min. All interviews were audio-recorded, transcribed verbatim and anonymized.

### Analysis

The data were analysed following the six steps for thematic analysis provided by Braun and Clarke [27] and their reflexive approach to thematic analysis [26]. Braun and Clarke [26] argued that a reflexive thematic analysis can be considered as the researcher’s interpretive analysis of data carried out at the intersection of data, theoretical assumptions and the researcher’s skills and resources. The authors have African (J.D.), Asian (S.A.) and European (M.F.) backgrounds, have academic training in nursing, physiotherapy and social sciences, and all participated in analysing and interpreting the data. J.D. has experience of being an immigrant and with gradual integration into Norwegian working life. During the participant interviews, he has been able more intuitively to capture and understand similar experiences and reflections described by HCWs and leaders, and thus pose in-depths and relevant follow up questions. S.A., as an academic immigrant worker, has contributed to validate the data analysis. Being born and educated in Norway, M.F. posed critical questions to fully understand the parts of the analysis work. In this manner, the various approaches have strengthened the analysis.

Initially, the transcripts were independently read by all the authors several times to gain familiarity and obtain a contextual impression. The material was also checked for readability and the reader’s ability to comprehend the text. We also noted our initial thoughts and impressions from the data. During the second phase, we reread the transcripts with a focus on the generation of initial codes relevant to the study’s research questions. The most relevant codes, in the form of words, sentences and paragraphs, were highlighted by each author. In this context, we were also aware of any basic underlying assumptions of the text, which were noted for discussion in later analyses. This phase took place in parallel with gaining insight into the concepts of ethnic diversity in healthcare and organisational culture and leadership. During the third phase, the codes assigned were reconsidered, and some were changed according to their relevance to different themes. Clarifying the complex relations between the codes and subcodes was challenging. Because of our different academic backgrounds and intercultural experiences, we encountered some differences in the coding of the data material, [28] but we were reflexive in our discussions so as to achieve richer interpretations of meaning [29]. The fourth phase included the division of themes, and the first common immediate interpretation concerned intercultural relations among HCWs, with ward nurses and patients at different levels at the nursing homes. The fifth phase included the redefinition of the names of the themes. This was carried out by reflecting on the themes, considering previous research and relevant concepts of organisational culture and leadership. This redefinition of the themes made them more relevant to the research questions and more comprehensive, enabling them to better elucidate the questions’ complexity. In the sixth and final phase of the analysis, we linked each theme to the most representative quotation to capture its essence and build a coherent narrative. A description of the analysis process related to one of the study themes is illustrated in Table 2.

**Table 2:** Example of the analyses process

Quotation	Initial codes	Subthemes	Themes
*We find it very exciting to work in a multicultural environment, and I have heard from others as well that it is very exciting. It’s both to know each other and to develop. There’s a difference between cultures, sometimes it can be a little difficult if you don’t know what’s common in your culture, but if you’ve worked with different people for a long time, you get used to it. It’s exciting to know about different countries, we taste food from different countries, and we help each other. (Participant 2)* *Those who have a multicultural background have a greater understanding, or if you have worked with multicultural people for a long time. We also had a leader who was Norwegian and who had worked with foreign people for a long time, and she had a lot of understanding; she understood the employees. You must work well together, understand them, have respect, and then you get it back. If you don’t respect and understand them, then you don’t get the help you require or, that work environment … It affects the working environment very much if you understand them. (Participant 1)*	Understanding across cultures Exiting with different cultures Multicultural background and diversity Respect for diversity Knowing the employees’ background	Greater understanding of diversity at work Shared experiences from multicultural environments	Understanding diversity through shared norms and multicultural experience

### Ethics

All participants took part in this study voluntarily and gave their written consent for the anonymised data to be used in publications. They were also informed that they could withdraw at any time during the study and that the data would be processed anonymously. The interviews were conducted in secluded offices, and the participants were informed about the anonymisation and protective storage of data. This study was approved by the Data Protection Official at the Norwegian Centre for Research Data (approval number: 689,018).

## Results

The analyses resulted in six themes: (1) understanding diversity through shared norms and multicultural experience, (2) greater flexibility in a multicultural workforce, (3) challenging traditional norms in a multicultural workforce, (4) language proficiency and exclusionary practices at work, (5) perceptions of the role of the ward nurse, and (6) prejudices among and harassment from patients.

### Understanding diversity through shared norms and multicultural experience

Most of the study participants found working in a nursing home ward with colleagues of different nationalities both exciting and instructive. Yet, it was the HCWs with minority backgrounds who commented to a greater extent on situations that were of importance to them as employees in a multicultural ward. Several people felt that a multicultural ward contributes to greater understanding across different cultural backgrounds. One of the nurses, for example, said that she has worked for a long time in environments where the HCWs have different backgrounds and that this made her more aware of various issues in the intercultural relations in the wards:

*Those who have a multicultural background have a greater understanding, or if you have worked with multicultural people for a long time. We also had a leader who was Norwegian and who had worked with foreign people for a long time, and she had a lot of understanding; she understood the employees. You must work well together, understand them, have respect, and then you get it back. If you don’t respect and understand them, then you don’t get the help you require or, that work environment … It affects the working environment very much if you understand them. (Participant 1)*

This nurse felt that awareness of important issues related to a multicultural working environment in the ward required experience with such work situations, but also characteristics such as respect and understanding. This is something that several of the ward nurses also highlighted when commenting on their experiences of running a multicultural ward. One of them explained that she arranges regular lunch meetings where ethnic minority HCWs can share about their background so that those in the ward can get to know each other better and gain a greater understanding of each other:

*I’ve found that you get a little deeper and better respect if you know a little bit about the background, and that’s why I’ve wanted to do it. I have received very positive feedback from those I have asked if they could tell me a little bit. It’s about 15 min, some people make more of it and some don’t make much of it, but just 15 min. Some will sit, some want to stand, some make PowerPoint slides. (Participant 15)*

Many of the study participants mentioned specific lunch days, for example, on Wednesdays or Fridays, when they came together and tasted food from different countries. They also marked the United Nations’ (UN) International Day in the workplace, when the HCWs could contribute food from their home country and perhaps also wear their national costume. Both the lunch days and the commemoration of the UN Day were considered important by staff and managers in contributing to greater transparency, greater understanding among staff and patients, and an increased sense of community.

### Greater flexibility in a multicultural workforce

The multicultural working environment created new opportunities for the accommodation of the HCWs’ preferences for working hours in nursing homes. Several had a cultural or religious affiliation, such that their holidays and the vacation needs were different from the majority population’s Christmas or Easter holidays. This created more room for flexibility in the organisation of the wards in particularly pressured seasons when HCWs were given days off depending on the time of year they celebrated their holidays:

*It’s about the operation of the ward. Those who have a different religious affiliation than Christianity, and if a deal has been made so that they can work several weekends and have a different holiday … Here, I would say that it is very narrow-minded; the Working Environment Act regulates this in a bad way. It assumes that everyone is Christian and is going to have Sunday free. It is a bit challenging when you have people with minority backgrounds and a different religion and a different holiday than what we have. (Participant 13)*

*We had an Eid celebration last time, and we have several Muslims working with us. All the Indians who worked switched with them. It was very nice that they respect that. (Participant 1)*

The ward nurses adhered to the working environment rules on holidays and work-free days but needed greater flexibility to ensure the continuous operation of the ward. This flexibility was possible because enough HCWs with minority backgrounds worked on several weekends and other holidays. Several of the HCWs also traded shifts among themselves so that their colleagues could celebrate their holiday. A multicultural workforce thus allowed a greater flexibility in the nursing homes and contributed to a smoother reshuffle of shifts between the HCWs.

### Challenging traditional norms in a multicultural workforce

Many of the HCWs with minority backgrounds found celebrating traditional holidays and the routines that followed in the nursing homes to be a major challenge. During holidays such as Christmas, Easter or the Norwegian national day, the norms and routines at the ward were changed for a period, and some HCWs lacked knowledge and experience with such routines. This often led to unfortunate situations in which the food service for the patients or other holiday traditions were not followed. One of the more experienced Norwegian-born auxiliary nurses described it as follows:

*As I said, we only have Norwegian residents, and they are in an age group where they have their habits and traditions. I understand that it is not so easy for everyone to familiarise themselves with all the Norwegian traditions, but we have experienced that custard has been poured on ribs on Christmas Eve! I have many times even offered, in conversation with the priest here at the house, to give instructions. For example, “What is May 17th? Why are we celebrating May 17th? What do we eat on May 17th and what do we do on May 17th?” The same for Easter and Christmas. The problem is that we have hectic working days, and it is difficult to get people to sit down and read. So, information, training, that’s what it’s about. (Participant 9)*

The HCWs with minority backgrounds often said that they had not grown up with Norwegian traditions and therefore did not have knowledge of the food traditions, manner of clothing or music associated with many of the holidays in Norway. Lack of knowledge or experience with such traditions was difficult for some:

*Yes, for example, for me Christmas is very difficult. The problem is that I do not know what the Norwegians sing, what food they eat, what they do, what clothes they wear. These things are very important, but often in our ward we have a Norwegian who takes responsibility; we go together. (Participant 3)*

In the wards, the staff on the shifts often consisted of both Norwegian-born and foreign-born HCWs, which allowed them to cooperate and help each other when challenges arose. Those who had knowledge of and experience with Norwegian traditions were often asked for advice or assistance. The ward nurses were also very concerned that all the HCWs should be able to do the job during the celebration of these holidays, and some said that they had initiated some measures to prepare them for important routines during the holidays:

*I want everyone here to know why we’re celebrating Easter, for example. There are also very few Norwegians who are born in Norway and are Christians who know why we celebrate Easter and know all the anniversary days of Easter. Therefore, I have often printed all these sheets for Easter … I also tell them: What day is this? Why is this a public holiday? I also want all employees to know this. I want them to bring it up with the residents because there are a lot of the residents who have a Christian view of life. (Participant 15)*

The ward nurses often prepared their own written guidance on the most important traditions, thus ensuring that the HCWs could perform well and that the patients had a good experience during the holidays. At the same time, both ward nurses and other HCWs said that there was a need to provide more structured training and an introduction to the most important holiday traditions.

### Language proficiency and exclusionary practices at work

Since the communication language with the patients in the multicultural ward was Norwegian, the working language was also Norwegian. Almost all the ward nurses and the other HCWs felt that the language competence of the HCWs with minority backgrounds was satisfactory for carrying out care work. They explained that nursing homes had introduced stricter language requirements for staff, which contributed to few HCWs having linguistic challenges:

*Those who work here, foreigners, are good at Norwegian, so there are no problems with residents there in relation to language … Because now, if you do not know proper Norwegian, there is something called a Norwegian test that you must take to be employed. But when I was at [the name of another nursing home] at the very beginning, I remember that we had some foreigners that were bad in Norwegian, and we noticed on the residents’ expressions. (Participant 8)*

Even though the participants felt that most of the health personnel had good knowledge of Norwegian, there could still be situations where more nuance was needed in the language, which could create challenges for some people. This at times meant that HCWs with minority backgrounds who felt less confident in the language were reluctant to initiate conversations with, for example, relatives who visited the patients:

*I think that we don’t really want to do it, but with communication, it ends up with the Norwegian speakers taking that bit the most. Those with minority backgrounds are a little reluctant, and it becomes easy to say: “Can you take it?” It gets challenging sometimes. We have someone who has worked here almost as long as I, but who still struggles a little with linguistics. But I also know that there may have been situations with relatives, for example, where they have given feedback that they did not fully understand correctly. (Participant 10)*

Although language was not seen as a major problem among the participants, a few occasions were brought up in which it caused difficulties. Yet it was unclear whether these incidents were real problems or whether they had something to do with the minority HCWs’ confidence in their language skills and the relatives’ and patients’ impatience with and reservations about the HCWs’ imperfect Norwegian.

Another issue highlighted by the multilingual staff was the need for HCWs to speak their native language with colleagues. For example, when two or more people who spoke a common language were gathered during lunch or in the dressing room, they changed from Norwegian to that language.

*Yes, we often use Norwegian, but sometimes when we work with someone from the same country, then we get the opportunity to use our language. We don’t meet very often, so when you meet, it’s freedom to talk about politics, other things. (Participant 3)*

Both ward nurses and other HCWs perceived it as “natural” that HCWs with minority backgrounds sometimes spoke in their native language during working hours. The most important thing was that they used Norwegian in the vicinity of the patients and other colleagues. However, some participants also reported that it was perceived as exclusionary when colleagues spoke a foreign language. Some felt “uncomfortable” not being able to understand what was being said, and this could lead to suspicion towards colleagues. However, everyone was aware of the importance of speaking the common language, Norwegian, and tried to avoid speaking another language in the common areas with several HCWs present. One of the ward nurses explained it this way:

*The common language at work is Norwegian, so we as an institution and The Nursing Home Agency are clear that it is the working language. This means that during working hours, they don’t speak native languages amongst themselves. They relate well to it. Sometimes they speak native languages when no one else is present, but as soon as someone else comes around, they turn to Norwegian. At least that’s my experience. (Participant 12)*

### Perceptions of the role of the ward nurse

Perceptions of and behaviour towards a ward nurse were issues in a multicultural ward that many of the HCWs and ward nurses highlighted. Some pointed out that a few of the HCWs were not used to shaking hands or having close contact with their ward nurses. HCWs also reported that some colleagues were reluctant to contact them in the department and often failed to express problems they faced or needs they had. One of the ward nurses reflected on the situation as follows:

*There are perhaps so many people from different cultures, and who themselves have told us that our view of a leader in our culture is almost … You don’t just go and talk to your manager in a way; you’re almost on guard instead. But that’s not my wish, at all, so they’re probably struggling a little bit with it. They do not seek me out– they do, but preferably not if they experience challenges, if there is a challenge they have, a work situation, or if there is a need one has about vacation, changing shifts, which one must talk to me about … Because we can talk about it. But their threshold for talking, just to have a chat, no more than that, is higher. (Participant 12)*

There was a common perception among the participants that some HCWs with minority backgrounds exhibited what they perceived to be more respect for leaders. This made them less inclined to seek out their ward nurse if needed. Some said that this was often a phase at the beginning of employment and that the HCWs gradually became more accustomed to the routines and work culture regarding the employee–leader relationship in the ward. For the ward nurses, the situation in which minority HCWs had a higher threshold for contact and expressing their views created a dilemma. Although they felt that respect was positive, it became more difficult to lead the personnel group when the HCWs did not contribute feedback about their work situation. Some of the ward nurses tried to reduce the HCWs’ threshold for contact through various measures. Some often left the office door open, or they attended the morning meetings of the HCWs more often and tried to spend more time in the ward to make themselves “harmless” as leaders.

At the same time, both ward nurses and other HCWs with a majority background expressed a great need for more knowledge about how they could utilise the multicultural resources but also include minority HCWs in the ward properly. One of the ward nurses explained what competence she would need in this way:

*While working with the whole personnel group, I’ve found out that things that many people think about, but that I have not ever thought of as a leader, can be perceived as a challenge. If something could be done to unite diversity, if one can use that word … (Participant 12)*.

Although many of the participants wanted more knowledge to understand the backgrounds of minority colleagues, they were also concerned that such a focus could lead to more stigmatisation of the group. Nevertheless, they wanted greater awareness of multicultural issues in nursing homes. The managers also expressed a need for competence in leading a multicultural personnel group, but they also said that HCWs should gain better knowledge about their rights and how to act in the work environment.

### Prejudices among and harassment from patients

All the HCWs reported that some patients had prejudices and spoke in an ugly way to the HCWs with minority backgrounds. Often, these were minor forms of harassment, but there could also be racist statements. One of the ward nurses recalled an episode in which an older patient refused help from an HCW with a dark complexion:

*We’ve had an example where a girl on the ward was thrown out of a lady’s room because the lady didn’t want that person in there. When we asked why it was like: “I don’t like the colour of her skin.” We didn’t go into any confrontation with the patient at the time, and I took the nurse aside and talked to her afterwards because I thought this might be a bad situation and this person can’t help the colour of her skin. It’s a racist remark, and I’m not going to defend it at all, but maybe explain why it happens. (Participant 13)*

One of the HCWs with a minority background talked about what patients usually said when presented with minority HCWs:

*Someone could be a little like this: Why are you here? We have some challenging patients, they react, they see that we are foreigners, and they can be very ugly in their mouths. “Go back to your country, what are you doing here?” It’s rare, but it happens. Even though we speak Norwegian, they think, “What kind of language do you speak?” Some have dialects, such as those from the Philippines or Thai. They have slightly different dialects. So, the patients react, and they sometimes get ugly in the mouths in front of employees. Not all of them, but there are some. (Participant 2)*

Although such situations did not arise daily, there were many episodes in which the participants or other colleagues with minority backgrounds experienced being verbally harassed. For example, they would receive remarks such as “I don’t want help by a black man”, “You are not a Norwegian nurse”, “I just want Norwegian.”

 The verbal scolding of minority HCWs was often categorised as racist, but most of it was assumed to be due to patients’ prejudices, age, lack of experience with foreign-born people or cognitive impairment. The participants explained that they had to distinguish between the person and the work situation and that they had to be professional. One of the HCWs with a minority background put it this way:

*If we get frustrated and a little annoyed, we must deal with it. We’re in a dementia ward and they’re sick, so we approach them and explain: “I work here, you live in a nursing home” and then, “I’m a nurse and you need my help.” (Participant 2)*.

Often, the staff managed to calm down the patients and finish their job. In some cases, they had to let another person take over, while the person with the minority background received emotional support from the ward nurse or colleagues. The participants felt that due to racist statements, they had to refuse patients the ability to choose from whom they would receive help. The patient and relatives who behaved in a racist way and who did not have cognitive impairment were spoken to and eventually refused the ability to change helpers.

 The problem of verbal harassment with racist undertones was known among all the ward nurses and HCWs. At the same time, some felt that the follow-up with the HCWs after such incidents was arbitrary. When asked if something systematic was done to remedy the situation, one of the nurses with a majority background answered as follows:

*Not enough. It’s easy for it to be just an incident, so it takes a long time for us to do something about it. I think in those situations, because there might be things that hurt, that you get distressed … So, taking things right away, then, “What did it do to you?” Right, “How did you feel?” (Participant 10)*.

This HCW felt that they spoke little about racially motivated harassment openly, even though everyone saw it as a serious problem for the wellbeing of minority HCWs in the ward. The topic was described as delicate, which contributed to a lack of focus on the problem.

## Discussion

The aim of this article was to explore how a multicultural workplace is experienced, through the accounts of HCWs and leaders in nursing homes. We highlight how increased ethnic diversity in nursing homes is experienced within the organisations’ cultural norms and practices. One of our main findings is that the HCWs from majority backgrounds, without prior experience of working in such settings, did not reflect much about diversity in their everyday work life as compared to HCWs from minority backgrounds. Prior experiences of working with ethnically diverse HCWs and getting to know ethnic minority HCWs’ backgrounds was perceived to facilitate better collaboration in the work environment. Thus, diversity was often discussed by HCWs in relation to celebrations such as UN Day or specific lunch days. Having marked days for common activities serves as a symbolic model for collective practices to unite HCWs and integrate them into a shared social and cultural sphere of meaning [30]. In doing so, these practices reflect the artefacts of organisational culture which may help nursing home wards to establish shared values in a multicultural workforce [21]. Such symbolic practices have also been found to create metaphorical spaces for negotiations and interpretations of identity for minority healthcare staff [31]. Therefore, how organisations connect with, engage and utilise people across differences can shed better light on the basic assumptions and values of the organisation with regard to diversity [21, 32]. Nevertheless, by limiting the understanding and experience of diversity to simply getting to know the HCWs’ “cultural background” and celebratory marked days/events, diversity can also risk becoming a token gesture or a symbolic offering in the workplace [33, 34]. In this context, Ahmed ([35], p. 605) argues that diversity can become “a politics of feeling good, which allows people to relax and think as if we have already ‘solved it’, and there is nothing left to do”. Of course, the problem is not the artefact of celebrating diversity in the multicultural wards. Rather, it is the lack of critical examination of basic assumptions in such working environments [21, 35].

Moreover, our findings indicate that while issues of staffing capacity in connection with holidays and weekends are a common challenge in nursing homes, several minority HCWs were able to take up work shifts during the majority population’s holidays. Thus, the presence of HCWs from diverse cultural backgrounds was perceived as a benefit of diversity and was reflected in the staffing practices employed in the organisation. However, this seemingly valuable aspect of diversity, i.e., minority HCWs’ different religious or cultural affiliations, also created some challenges in the nursing homes. Such challenges were discussed primarily in reference to minority HCWs’ limited knowledge of Norwegian cultural traditions. Considering that living in a care setting such as a nursing home can be quite a significant change in the life of older residents [36, 37], it is undoubtedly important to be able to ensure security and comfort through a familiar environment [38, 39]. For the HCWs to be able to carry out their work skilfully and always have relevant expertise, the management must ensure that the HCWs have up-to-date knowledge and practical training [40], which could include cultural customs in a Norwegian nursing home context.

Although language problems among the minority HCWs were widely dismissed, our findings show that some patients and relatives still complained. This could serve as an example of an exclusionary practice in the multicultural workforce, especially if it is not addressed properly – if it is just taken for granted that HCWs of majority background can step in and take over. Appeasing the patient and relatives at the expense of the minority HCWs, as it may seem, underestimates the underlying processes of subtle discrimination in the workplace driven by a power process that has a disempowering impact on those exposed to this type of behaviour [41]. Awareness of such taken-for-granted behaviour and hidden assumptions may impede the cohesion of the organisational culture of nursing homes and compromise the wellbeing of minority HCWs.

Language is an integral aspect of organisational culture. Our study found that all HCWs acknowledged the importance of maintaining Norwegian as the common language, both when talking to and within the vicinity of residents. However, linguistic diversity was also found to become the basis for cohesiveness among minority HCWs, as they were able to talk amongst themselves in their native language and have a common cultural context. Nevertheless, in some situations, this practice was perceived as exclusionary for HCWs from a majority background who did not understand the language. Linguistic diversity in organisational culture can thus create possibilities of both solidarity and exclusion [42]. Nursing homes need, therefore, to be aware of language use’s implications for group cohesion in the multicultural workforce.

Our findings also showed that the HCWs with minority backgrounds faced challenges that the management was not always aware of. Often, these challenges were related to the HCWs’ misinterpretation of a manager’s role in a Norwegian context. This was demonstrated by HCWs’ hesitance to contact managers. It is not uncommon for expectations to exist about the relationship between the manager and the employee of another country’s organisational culture [33, 43]. HCWs’ hesitation to contact managers was discussed by Minimoshvili and Cerne [44], who claimed that such behaviour by minority members occurs as a reaction to being perceived as different and to their experience of not being included in the culturally dominant work community. Leaders play a central role in defining and maintaining organisational culture [45]. A leader must be able to understand the employees’ problems and challenges in the workplace to manage them appropriately. This is even more important when differing cultural perceptions prevail [46]. Schein [45] suggested that leaders must develop clarity about the organisational climate and culture they hope to promote, which would be necessary for the HCWs in nursing homes to be able to understand their role and that of the managers in a more conducive environment that supports learning and sharing of cultural perceptions at work. For organisations to function efficiently, managers must create an inclusive working environment that considers the needs of all employees [43]. Managers’ lack of knowledge or awareness of the distanced manager-employee relationship may therefore create challenges in obtaining relevant information from the minority HCW and in maintaining a well-functioning multicultural working environment. Managers need to make it comfortable and safe enough to talk about cultural differences and similarities in the nursing home workforce [47]. Such measures may contribute to a safer working environment for HCWs and staff so they can provide better services for patients.

Discrimination and harassment based on ethnic affiliation are serious working environment problems. They reduce workers’ psychosocial wellbeing and is something leaders are expected to prevent [48]. We found that racism towards minority HCWs by residents often occurred. Discrimination against HCWs while they are carrying out their work is well documented, both in Norway and internationally [13, 49–52]. For example, a study on nursing homes in Norway found that ethnic minority workers, particularly those with a darker skin tone, were subjected to racist remarks from patients [13]. Similar findings emerged from a study of nursing homes in Sweden, where ethnic minority HCWs were not only subjected to racism but were also less respected by both residents and their families, compared to native HCWs [51].

However, what makes the issue of racism challenging to tackle in the context of nursing homes is the fact that many of the residents often suffer from cognitive impairments. The politics of diversity is complex and contextual [53]. Racism, especially when intertwined with illness, has complex ethical and medical/care implications, as minority HCWs do not have the opportunity to respond or cope in ways they could if the incident had occurred in a different context [52]. Although the participants in our study rationalised such encounters and showed understanding, such incidents can nevertheless have a psychological and emotional impact [54–57]. They can also lead to withdrawal and disengagement from work [58] and thereby impact the external health of the organisation. In this regard, Philomena Essed argued that the discourse of discrimination can make those who experience it into the problem [59]. The minority staff likely find it challenging to talk about their experience of discriminatory encounters out of the concern that they will be perceived as those who pose the problem. Thus, such incidents pose a challenge to both the internal and external health of the organisation, with consequences to the wellbeing of the HCWs in a multicultural workforce.

In our study, it seems that patients referred to the same prejudices and harassment that abound in the debates about immigrants in the media [60]. Thus, this is not merely an individual problem, but a serious social problem that involves the use and abuse of power with far-reaching negative consequences for those exposed to racism [61]. Therefore, it is important to recognise that organisational cultures are nested in larger societal cultures [21]. Snyder and Schwartz [54] discussed how minority HCWs may experience racism in other contexts as well, which can result in compounded stress, which in turn may further impact their mental and physical health [62]. Systematic follow-up of such incidents by leaders is thus vital. Most of the participants reported that such incidents were not discussed later or followed up on, with the assumption that they had been handled. This is understandable, given that diversity work means working with problematic terms [53], and leaders may find it challenging to broach such topics in both formal and informal settings. However, when such incidents are left unaddressed, they are construed as unproblematic and normal [63]. The organisations’ underlying conflicts and power structures become repressed and construed as normal. Thus, there seems to be a need for formal processes to deal with such incidents, which include, but are not limited to, discussions with the affected HCW and discussions with the team to share and learn from such experiences. It is important for the handling of such incidents to reflect in both the espoused values and the underlying assumptions within the culture of the nursing homes.

Considering that a larger proportion of older people in society will trigger a need for more HCWs in nursing homes, persons with immigrant backgrounds will undoubtedly be a major asset in this regard [64]. It is therefore important to ensure good working conditions and competent and engaged management that understands the challenges of HCWs with minority backgrounds. Research in personnel and human resources management has stated the importance of seeking ways to fully take advantage of the benefits of diversity [65]. To this end, to ensure the internal cohesion of the organisation [21], nursing homes should aim at promoting inclusion, which serves as a key to gaining the benefits of diversity. Inclusion involves “how well organisations and their members fully connect with, engage, and utilise people across all types of differences” ([32], p. 4). The HCWs’ wellbeing, and subsequent enhanced delivery of care to a growing older patient population, depends highly on the commitment of the nursing homes’ management to inclusion for all their HCWs [66], which is especially urgent in dealing with an ethnically diverse work environment.

### Strengths and limitations

Several factors contribute towards the trustworthiness of this study. Although the study participants were recruited through contact with each nursing home and its managers, by reminding them that the interviews were confidential, we were able to obtain interesting and unexpected data, especially on the topic of prejudice and discrimination. In addition, the interviewer’s migrant background and experience in municipal care research seemed to help make the study participants comfortable enough to engage in meaningful dialogue. Further, all three authors were involved in the analysis and interpretation of findings, thus facilitating multiple ways of seeing the data and addressing any possible researcher bias. This also helped to ensure a good balance of closeness and analytical distance to the material [67]. Additionally, the inclusion of participants’ verbatim quotes and previous research highlights the analytical process and substantiate our findings, respectively [68].

Regarding transferability, our study provides a wide range of participants’ perspectives as well as contextual information to enable thick description and meaningful interpretations [67, 69]. However, since we only conducted individual interviews, this may have limited understanding about their responses in a group setting. Focus group discussions on similar institutions in other parts of Norway and internationally are therefore recommended.

### Implications for practice

Our findings indicate that there is a need for increased knowledge and awareness of how multicultural work communities in nursing homes function. To equip HCWs with a minority background with up-to-date competence, the employer should ensure that they are introduced to the central cultural traditions that patients value, as well as provide language support where necessary. Managers should raise their awareness of leading multicultural wards and know their employees’ situations and varied needs in working life. Importantly, they must prevent harassment in the ward by taking patients’ racism seriously, as it can have major consequences for the wellbeing of HCWs and their functioning in the workplace.

## Conclusions

With an increasing proportion of older people needing care in nursing homes, and increased recruitment of health personnel is necessary. Diversity in the health professions is therefore paramount to the capacity of the nation’s healthcare system to meet the needs of the ageing population. If people with immigrant backgrounds are to be able to provide competent help and thrive, the sector must understand that mere diversity in numbers is not sufficient. HCWs must also feel included in that the working environment is adapted to their need for increased competence and wellbeing. Diversity in the nursing workforce must therefore be acknowledged beyond mere representation. To ensure an effective organisation, managers must develop an inclusive organisational culture, and they must be able to engage with difficult topics and conflicts that may arise in a multicultural workforce. This will create a safe working environment, increase HCWs’ ability to be innovative and boost the organisations’ delivery of enhanced care for all patients.

## Data Availability

The data that support the findings of this study are available upon request from the corresponding author. The data are not publicly available due to information that could compromise the privacy of the research participants.
